# Editorial: Classical and Modern Biotechnology Applied to the Treatment of Epilepsy and Anxiety Disorders

**DOI:** 10.3389/fphar.2021.824986

**Published:** 2022-01-14

**Authors:** R. O. Beleboni, M. R. Mortari, L. Melo-Thomas, N. C. Coimbra

**Affiliations:** ^1^ Biotechnology Department, School of Medicine, University of Ribeirão Preto, Ribeirão Preto, Brazil; ^2^ IB-CFS, University of Brasilia, Brasilia, Brazil; ^3^ Philipps-Universität Marburg, Marburg, Germany; ^4^ FMRP-USP, University of São Paulo, Ribeirão Preto, Brazil

**Keywords:** comorbidities, clinical management, mood disorders, seizures, TLE

The classical and modern approaches applied to the search for new therapeutic options in neurological disorder management have rendered important theoretical and practical innovations, especially when considering the broad contribution of biotechnology advances (Maguire, 2019). Our research topic dealing with these issues was carefully assembled by reviews and original research articles written by invited eminent researchers from different countries in which neurological disorders are equally relevant and customarily represent a huge public health problem. This statement is essentially true when those disorders appear as comorbidities such as in the case of epileptic patients with anxiety and depression as examples of more common comorbidities. As supported by solid clinical evidence, it has been assumed that the improvement or worsening of symptoms elicited by depression and/or anxiety may produce respectively a positive or negative influence on the prognosis of epileptic condition and *vice versa* (Thapar et al., 2009). Several different contributing factors can modulate the neuropathology and prognosis underlying different neurological disorders *per se*. However, and despite the important molecular and cellular bases differences in those disorders, the sharing of specific pathophysiological mechanisms and neural pathways subserving epilepsy and simultaneously involved in the regulation of behavior and mood underlying anxiety or depression allows the use of combined therapeutic strategies for each specific case (Thapar et al., 2009; Dhir, 2010).

The significant symptom relief and/or cure for the patients suffering from those neurological conditions separately or in comorbidities with psychiatric diseases is the golden goal for clinicians and pharmacologists, each time mostly aided by biotechnological advances (Maguire, 2019). The most imperative advances should be focused on avoidance of side effects elicited by the current arsenal of drugs, as well as in the shortness of time for the onset of their pharmacological action when applied for the treatment of those medical conditions. In addition, the increase of therapeutic compliance of patients and a satisfactory therapeutic response to the novel therapeutic strategies, especially when applied in therapy of refractory cases, are equally welcome (Beyenburg et al., 2005; Maguire, 2019). Based on this, new theoretical and practical therapeutic innovations, the earlier and more correct diagnosis, a more complete and clear nosological classification, and an advanced understanding of different aspects of those neurological disorders are particularly expected and worthy (Beyenburg et al., 2005; Thapar et al., 2009; Dhir, 2010; Maguire, 2019).

In this context, a mini-review article by Operto et al. highlights several technical details about the use of ketogenic diet in the alternative and/or complementary management of epilepsies in children and adolescents with a higher risk of mood disorders. The incidence and/or prevalence of mood disorders in pre-adolescents or adolescents is higher than in other age counterparts of the general population, and anxiety and depression are highly prevalent in children or adolescents with newly diagnosed epilepsy when compared to healthy young people. The critical conditions of mood disorders and the types of epilepsies, in which the most common version of the ketogenic diet exhibit the best therapeutic response, are considered in this mini-review. Briefly, ketogenic diets may modulate the functioning of different classical monoaminergic and GABA/glutamatergic neurotransmission, mitochondrial function, and oxidative stress balance of neuronal cells with different mechanistic impacts. Therefore, a more complete understanding and/or development of new ketogenic diet variants, in which a higher patient tolerability and acceptance are archived, as well as those with increased therapeutic efficacy, particularly in the epilepsy refractory cases, is imperative.

Still, Zhu et al. contributed an original research article, in which the therapeutic potential of 5-HT_6_ serotonergic receptor signaling modulation was investigated by use of the well-established pilocarpine-induced epilepsy model in rats. A combined set of electrophysiological and molecular experiments was used to investigate the role of 5-HT_6_ serotonergic receptor ligand-induced mechanistic changes in the long-term potentiation (LTP) *via* the KCNQ_2_ potassium channel M-current regulation. More precisely, the blockade of 5-HT_6_ receptors by SB-271046 was shown to reduce the pilocarpine-induced LTP impairments by regulating the KCNQ_2_-mediated M-currents in rats. As a seminal conclusion, it was shown that the 5-HT_6_ receptor blockade decreases the excitability of the hippocampal pyramidal neurons in pilocarpine-induced status epilepticus in rats by an improved regulation of inhibitory and excitatory neurotransmission balance, with a significant and potential impact for the further advances in the treatment of epilepsies.

Also, using pilocarpine-induced status epilepticus, Kohek et al. have written an original research paper in which the influence of different anxiety conditions in the temporal lobe epilepsy and immunohistological modifications on interconnected brain areas were investigated regarding different aspects of epileptogenesis. Neuronal loss and the expression of selective neuromodulators, such as neuropeptide Y, in rodent hippocampus and amygdala, were finely studied with relevant considerations about common and potential therapeutic targets to better management of both disorders.

Parvalbumin inhibitory interneurons are strictly involved in different neural pathways activity, as well as in neurometabolic mechanisms underlying the onset and maintenance of different brain disorders, in which mood regulation is substantially required. In a systematic review and in a subsequent meta-analysis, Pinna and Colasanti showed evidence about the reciprocal relationship between the particular vulnerability of parvalbumin interneurons to metabolic stress and the newly associated mitochondrial dysfunction in affective disorders. The role of those interneuron losses or dysfunction/downregulation on mood instability of excitability states in bipolar affective disorder was discussed. Finally, the potential pharmacological targeting of parvalbumin-labelled interneurons was suggested as a new strategic avenue in the management of psychiatric and neurological conditions, in which the cyclic neurometabolic and neuroexcitability abnormalities promoted by the losses or downregulation of those inhibitory cells have been determined.

Finally, considering the diversified contribution of articles published here, we expected to motivate researchers to explore new horizons focusing on new approaches aiming a better understanding of epilepsy in comorbidity with mood disorders expecting a safe and most effective treatment of these disorders, complying with the improvement of the overall quality of life of affected patients.
